# Impact of reduced ignition propensity cigarette regulation on consumer smoking behavior and quit intentions: evidence from 6 waves (2004–11) of the ITC Four Country Survey

**DOI:** 10.1186/1617-9625-11-26

**Published:** 2013-12-21

**Authors:** Sarah E Adkison, Richard J O’Connor, Ron Borland, Hua-Hie Yong, K Michael Cummings, David Hammond, Geoffrey T Fong

**Affiliations:** 1 Department of Health Behavior, Roswell Park Cancer Institute, Elm & Carlton Streets, Buffalo, NY 14263, USA; 2 The Cancer Council Victoria, Melbourne, Victoria, Australia; 3 Department of Psychiatry and Behavioral Sciences, Medical University of South Carolina, Charleston, SC, USA; 4 School of Public Health and Health Systems, University of Waterloo, Waterloo, ON, Canada; 5 Department of Psychology, University of Waterloo, Waterloo, ON, Canada; 6 Ontario Institute for Cancer Research, Toronto, ON, Canada

**Keywords:** Reduced ignition propensity, Fire-safe cigarettes, Consumer perceptions, Generalized estimating equations (GEE)

## Abstract

**Background:**

Although on the decline, smoking-related fires remain a leading cause of fire death in the United States and United Kingdom and account for over 10% of fire-related deaths worldwide. This has prompted lawmakers to enact legislation requiring manufacturers to implement reduced ignition propensity (RIP) safety standards for cigarettes. The current research evaluates how implementation of RIP safety standards in different countries influenced smokers’ perceptions of cigarette self-extinguishment, frequency of extinguishment, and the impact on consumer smoking behaviors, including cigarettes smoked per day and planning to quit.

**Methods:**

Participants for this research come from Waves 3 through 8 of the International Tobacco Control (ITC) Four Country Survey conducted longitudinally from 2004 through 2011 in the United States, United Kingdom, Australia, and Canada.

**Results:**

Perceptions of cigarette self-extinguishment and frequency of extinguishment increased concurrently with an increase in the prevalence of RIP safety standards for cigarettes. Presence of RIP safety standards was also associated with a greater intention to quit smoking, but was not associated with the number of cigarettes smoked per day. Intention to quit was higher among those who were more likely to report that their cigarettes self-extinguish sometimes and often, but we found no evidence of an interaction between frequency of extinguishment and RIP safety standards on quit intentions.

**Conclusions:**

Overall, because these standards largely do not influence consumer smoking behavior, RIP implementation may significantly reduce the number of cigarette-related fires and the associated death and damages. Further research should assess how implementation of RIP safety standards has influenced smoking-related fire incidence, deaths, and other costs associated with smoking-related fires.

## Background

Although on the decline, smoking-related fires remain a leading cause of fire death in the United States (US) [1] and United Kingdom (UK) [2] and account for over 10% of fire-related deaths worldwide [3]. This has prompted lawmakers to enact legislation requiring manufacturers to implement reduced ignition propensity (RIP) standards for cigarettes. These cigarettes are [4] sometimes inappropriately called “fire-safe” because the standards only reduce the risk of fire. RIP cigarettes are designed with paper that has lower porosity bands, which are designed to self-extinguish when not being continually smoked [5].

Attempts to legislate the manufacturing of RIP cigarettes began in the US in 1974, although the first law was not implemented until 2004 in New York State [6]. Additional states followed suit thereafter (http://www.nfpa.org). In October 2005, Canada became the first country to mandate RIP cigarettes (Bill C-260) [7] and many other countries and jurisdictions have also done so, including Australia in March 2010 [8]. In the US and Canada the legislation requires that all cigarettes manufactured burn their full length no more than 25% of the time when evaluated with the American Society for Testing Materials (ASTM) International method E2187-04: Standard Test Method for Measuring the Ignition Strength of Cigarettes [9]. In the US, all cigarettes that comply with the policy have packs that are labeled with “FSC” (Fire Standards Compliant) above the UPC to note that they meet RIP standards.

The tobacco industry historically opposed RIP legislation, although it has recently expressed greater support [10]. Some of the industry arguments against RIP cigarettes centered on the allegedly increased toxicity and reduced consumer appeal [11,12]. Reduced consumer appeal, they argued, might result in changes in smoking behaviors including intentions to quit and quitting altogether [13,14] because cigarette design features influence the consumer experience [15]. There is also evidence RIP cigarette safety standards increase the odds of perceptions and frequency of cigarette self-extinguishment [16-18]; however, this has not been associated with significant changes in smoking behaviors or in intention to quit [16,18]. No research to date has assessed impacts of RIP standards on smoking behaviors in the longer term beyond implementation. The present research evaluates the impact of RIP safety standards over 6 waves of data from the ITC 4 Country survey to assess the impact on smoking behaviors associated with implementation over the medium term.

Specifically, the current research evaluates how implementation of RIP cigarette regulations in different countries influenced smokers’ perceptions of cigarette self-extinguishment, frequency of extinguishment, and the impact on consumer smoking behaviors including number of cigarettes smoked per day and intention to stop smoking.

## Methods

Participants for this research come from Waves 3 through 8 of the International Tobacco Control (ITC) Four Country Survey (ITC-4) conducted longitudinally from 2004 through 2011 in the United States (US), United Kingdom (UK), Australia, and Canada. The ITC-4 began in 2002 and includes a longitudinal cohort design with a sample of approximately 1,500-2,000 adult smokers in each country who have been interviewed approximately annually about their smoking behavior. At initial enrollment, respondents included smokers aged 18 or older who smoked at least 100 cigarettes in their lifetime and at least 1 cigarette in the past 30 days. Probability sampling methods were used to recruit the sample using a random-digit dialing technique. If multiple adult smokers were present in the home, the next-birthday method was used to select the respondent. Additional respondents were recruited yearly to replenish the cohort for those lost to attrition. Greater details regarding the study design, sampling frames, and overall aims of the project are described elsewhere [19,20]. This study was approved by the Research Ethics Board of the University of Waterloo and the Institutional Review Board of Roswell Park Cancer Institute.

### Measures

The primary independent variable of interest was the presence or absence of an RIP law. RIP was coded as a dichotomous variable at each time point to indicate whether or not an RIP law was in place for each respondent at the time of the survey. In the US, implementation of RIP standards varied by state beginning at Wave 3(http://www.nfpa.org). In Canada a federal law was implemented just prior to Wave 4 on October, 1 2005 [7] and in Australia a federal law took effect prior to Wave 8 on March, 23 2010 [8]. There was no law requiring the use of RIP paper for cigarettes in the UK during the study period. Table 1 presents the percent of respondents in each country subject to RIP safety standards at each Wave:

**Table 1:** Percent of respondents in each county in jurisdictions with RIP cigarette safety standards

**Fieldwork date**	**US**	Canada^*^	**Australia**	**UK**
Jun 2004 – Dec 2004 (Wave 3)	5.4	0	0	0
Oct 2005 – Jan 2006 (Wave 4)	7.3	100	0	0
Oct 2006 – Feb 2007 (Wave 5)	11.9	100	0	0
Sep 2007 – Feb 2008 (Wave 6)	23.7	100	0	0
Jun 2009 – Dec 2009 (Wave 7)	45.7	100	0	0
Jul 2010 – Jun 2011 (Wave 8)	96.8	100	100	0

*Implementation began only 13 days prior to the start of the Wave 4 survey in Canada.

To assess the impact of the RIP law on perceptions of self-extinguishment the dependent variables assessed were reported awareness of and frequency of cigarette self-extinguishment between puffs. Participants who indicated that they currently smoked were asked, “Do your cigarettes ever go out between puffs?” If the respondent said “yes”, the follow-up question “How often?” was asked to assess the frequency of extinguishment. Response options included: rarely, sometimes, and often. Smokers who reported that their cigarettes did not go out were coded as “Never”. This was converted to a dichotomous variable representing self-extinguishment “often” and “less than often.”

To assess whether implementation of RIP safety standards for cigarettes influenced smokers’ cigarette use and quit intentions we evaluated changes in the number of cigarettes smoked per day and intentions to quit smoking (Yes, No).

### Statistical analyses

Data were analyzed using SPSS 21.0 (SPSS, Inc., Chicago, IL). Generalized estimating equations (GEE) were used to evaluate the impact of RIP laws on perceptions of cigarette self-extinguishment and smoking behaviors. The GEE method has several advantages for analyzing longitudinal data because it uses all available data points, provides a method for handling the correlated nature of repeated measurements, and accounts for the pattern of change over time. A binomial distribution with logit link function was employed to evaluate the dichotomous dependent variables. We selected a first-order autoregressive correlation structure because two observations taken closer in time within an individual are likely to be more highly correlated than two observations taken further apart in time. Shults et al. (2009) [21] illustrated that this structure is appropriate for binary longitudinal data. We used the power (0.5) link function for the analysis of cigarettes smoked per day to account for the distribution of the dependent variable.

Covariates in the analyses included age (18–25, 26–40, 41–54, 55+), race (white, non-white), sex (male, female), education (Low = high school or less, Moderate = some technical school or some university, High = University degree or more), income (low = less than 30,000 USD, moderate = 30,001-59,999, high = 60,000+), cohort, and cigarettes smoked per day when appropriate.

## Results

### Participants

Table 2 displays the distribution of respondents by demographic variables at each wave. Overall, there were 12,492 smokers of entirely or predominantly factory made cigarettes and a total of 33,089 observations. Mean age of the sample for observations was 43.2 years (sd: 14.26); over half were female (50.8%), 28.9% were from the US, 26.3% from Canada, 24.4% from Australia, and 20.4% from the UK. There were fewer respondents in the UK because prevalence of roll your own cigarette is much higher in this country [22,23]. Respondents who smoked primarily RYO were eliminated from this analysis because those cigarettes are not subject to the RIP safety standards.

**Table 2:** **Sample distribution by demographic variables**^**1 **^**for each wave**

	**Wave 3**	**Wave4**	**Wave 5**	**Wave 6**	**Wave 7**	**Wave 8**
	**N = 6423**	**N = 5874**	**N = 5815**	**N = 5650**	**N = 4694**	**N = 3530**
Sex						
Female	50.6	50.7	51.1	50.7	50.7	51.1
Male	49.4	49.3	48.9	49.3	49.3	48.9
Country						
US	28.5	28.3	28.2	27.5	27.7	30.3
Canada	25.9	25.8	25.9	26.7	27.6	29.5
Australia	24.5	24.7	25.7	26.2	23.8	24.4
UK	21.1	21.1	20.2	19.5	20.9	15.8
Age						
18-25	10.5	10.7	11.5	12.9	12.0	12.4
26-40	29.6	28.8	29.4	28.1	27.3	25.2
41-54	36.8	37.3	36.9	37.9	39.6	41.2
55+	23.1	23.2	22.1	21.1	21.1	21.2
Race^2^						
White	87.6	88.1	87.9	88.0	88.9	89.6
Non-White	12.4	11.9	12.1	12.0	11.1	10.4
% RIP Standards	1.5	27.9	29.2	33.3	40.2	83.3

Note: ^1^, distribution based on the dependent variable do your cigarettes self-extinguish. Numbers shift slightly with other dependent variables; ^2^, n is slightly less because some respondents did not provide race data.

### Perceptions of cigarette self-extinguishment

**Figure 1:**
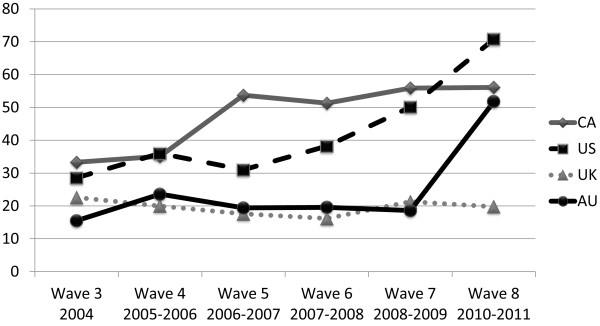
Percent of respondents reporting their cigarettes self-extinguish by country.

Figure 1 depicts the percent of respondents who reported that their cigarettes self-extinguish at each wave, by country. Noticing self-extinguishment largely mirrored the pattern of implementation within each country (see Table 1). In the US, an increasing proportion of smokers noticed that their cigarettes self-extinguished over time, consistent with the gradual increase in the number of states that enacted RIP laws. In Canada, where the RIP law was enacted shortly prior to W4 fieldwork, reported cigarette self-extinguishment changed little from baseline (Wave 3) at Wave 4; however, by Wave 5, reported self-extinguishment rose from 37% to 54%, corresponding with the full compliance with the RIP law. In Australia, awareness of extinguishment rose from 29% to 57% between Wave 7 and Wave 8, corresponding to implementation of RIP standards. In the UK, where no law was in place, the percent reporting that cigarettes self-extinguish remained consistently low. At Wave 8 nearly three-quarters of respondents in the US reported that their cigarettes self-extinguish, significantly higher than in other countries with RIP safety standards (CA and US: χ^2^ (N = 2097, 1) = 48.397, p < .001; AU and US: χ^2^ (N = 1919, 1) = 72.559, p < .001.

Table 3 outlines the results of the GEE analysis. Respondents with an RIP law in place had nearly 3 times greater odds of reporting that their cigarettes self-extinguished (Model 1). Smoking more cigarettes per day was associated with increased odds of noticing self-extinguishment. In addition, an interaction was present between study wave and country for the United States and Canada. Within country analyses revealed that, in Canada, respondents had 2 times greater odds of noticing self-extinguishment beginning at Wave 5 through Wave 8 than compared with Wave 3. In the US, compared with Wave 3 respondents had greater odds of noticing self-extinguishment during Waves 4, 7, and 8 corresponding to Figure 1. As expected, there was no association between wave and self-extinguishment among UK respondents.

**Table 3:** Generalized estimating equation analyses for perceptions of RIP cigarettes

		**Model 1**			**Model 2**		
		**Do your cigarettes go out?**			**Do they go out often?**		
		**B (se)**	**OR**	**95% CI**	**B (se)**	**OR**	**95% CI**
**RIP Standard**	Law in Place	1.007(0.043)	2.737***	2.516-2.978	1.109(0.080)	3.030***	2.593-3.542
	No Law		REF			REF	
**Country**	United States	0.414(0.091)	1.513***	1.267-1.807	-0.353(0.174)	0.703*	0.500-0.988
	Canada	0.241(0.093)	1.273**	1.060-1.528	-0.326(0.178)	0.722+	0.509-1.024
	Australia	0.260(0.100)	1.297**	1.071-1.571	-0.268(0.198)	0.765	0.518-1.128
	United Kingdom		REF			REF	
**Wave**	Wave	0.020(0.021)	1.021	0.980-1.063	-0.016(0.039)	0.984	0.912-1.062
Country*Time	United States	0.113(0.025)	1.119***	1.065-1.176	0.228(0.048)	1.256***	1.143-1.379
	Canada	0.067(0.026)	1.069**	1.017-1.124	0.083(0.045)	1.087+	0.994-1.188
	Australia	0.004(0.026)	1.004	0.954-1.058	0.075(0.053)	1.078	0.972-1.195
	United Kingdom		REF			REF	
**Cigarettes Per Day**	CPD	0.003(0.001)	1.003*	1.000-1.006	0.002(0.002)	1.002	0.998-1.007
**N**			12,492			12,483	
**Observations**			33,089			33,050	

Note: ***p<.001, **p<.01, * p < .05, +p < .10, models were adjusted for age, sex, education, income, ethnicity, and cohort.

**Figure 2:**
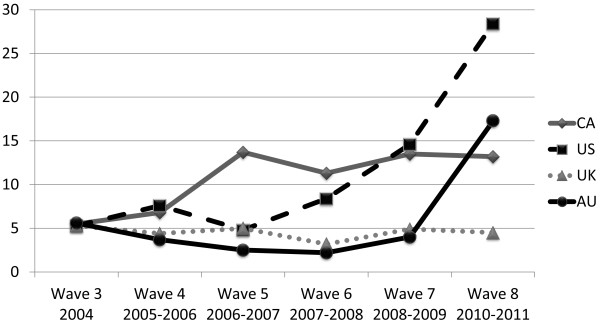
Percent of respondents reporting their cigarettes self-extinguish “often” by country.

Figure 2 depicts the percent of smokers in each country reporting that their cigarettes self-extinguish “often.” Similar trends are present mirroring the implementation of RIP safety standards. Again, during Wave 8, smokers in the US were significantly more likely than smokers in the other countries to report that their cigarettes self-extinguish often (CA and US: χ^2^(N = 2095, 1) = 73.087, p < .001), AU and US: χ^2^(N = 1916, 1) = 32.665, p < .001).

Model 2 estimates the odds of RIP laws impacting perceptions of the frequency that cigarettes self-extinguish often. Where laws were in place, the smoker population had 3 times greater odds of reporting that cigarettes self-extinguish often. An interaction between the US and Wave was present. And within country analyses showed, that, in the US, respondents had greater odds of reporting self-extinguishment often at Waves 4, 6, 7, 8. In Canada, beginning at Wave 5 respondents had significantly greater odds of reporting self-extinguishment often as compared to Waves 3 and 4.

### Cigarette consumption and intentions to quit smoking

Models 3 and 4 (Table 4) present the GEE analyses for the impact of RIP standards on the number of cigarettes smoked and intention to quit smoking. RIP safety standards were not significantly associated with changes in the number of cigarettes smoked per day. Furthermore, the frequency of self-extinguishment was not associated with cigarette consumption per day. Respondents were more likely to smoke fewer cigarettes at each time point following the enrollment. RIP safety standards were, however, significantly associated with increased odds of intending to quit smoking. Additionally, reporting that cigarettes self-extinguish sometimes or often was associated with a greater intention to quit. These models were also adjusted with sociodemographic correlates of cigarette use.

**Table 4:** Generalized estimating equation analyses for changes in smoking behavior

		**Model 3**			**Model 4**		
		**Cigarettes per day**			**Intention to quit**		
		**B (se)**	**OR**	**95% CI**	**B (se)**	**OR**	**95% CI**
**RIP Standard**	Law in Place	0.011(0.018)	1.0011	0.975-1.048	0.018(0.008)	1.018*	1.003-1.034
	No Law		REF			REF	
**Time**	Wave	-0.066(0.005)	0.936***	0.926-0.944	-0.010(0.002)	0.990***	0.986-0.994
**Country**	United States	0.043(0.016)	1.044*	1.012-1.078	0.050(0.010)	1.051***	1.031-1.072
	Canada	0.003(0.022)	1.003	0.960-1.047	0.096(0.011)	1.101***	1.077-1.125
	Australia	0.036(0.021)	1.037+	0.996-1.080	0.100(0.010)	1.106***	1.084-1.127
	United Kingdom		REF			REF	
**Frequency of**	Often	-0.039(0.023)	0.962	0.920-1.005	0.020(0.009)	1.020*	1.002-1.039
**Self-extinguishment**	Sometimes	0.008(0.018)	1.008	0.974-1.044	0.027(0.006)	1.027***	1.014-1.040
	Rarely	0.015(0.015)	1.015	0.986-1.045	0.007(0.008)	1.007	0.991-1.023
	Never		REF			REF	
**Cigarettes Per Day**	CPD at enrollment	0.074(0.004)	1.077***	1.069-1.085	--	--	--
**Cigarettes Per Day**	CPD	--	--	--	-0.004(0.000)	0.996***	0.995-0.996
**Intend to Quit**	Yes	-0.098(0.011)	0.907***	0.888-0.926	--	--	--
	No		REF		--	--	--
**N**			12,388			12,388	
**Observations**			32,536			32,536	

Note: ***p<.001, **p<.01, *p < .05, +p < .10, models adjusted for age, sex, education, income, ethnicity, and cohort; CPD at enrollment is modeled to assess overall CPD overtime while CPD at each wave is modeled to evaluate intention to quit smoking overtime.

## Conclusions

Perceptions of cigarette self-extinguishment and frequency of extinguishment increased concurrently with an increase in the prevalence of RIP safety standards for cigarettes. The presence of RIP safety standards did not have an effect on the number of cigarettes smoked per day. Intention to quit was higher among those who were more likely to report that their cigarettes self-extinguish sometimes and often, though the effect size was quite small, and there was no evidence of an interaction between frequency of extinguishment and RIP safety standards on quit intentions. Perhaps those who are thinking more intently about changing their smoker status are also more likely to notice changes in product performance because they are already dissatisfied with their smoking behavior.

While trends in noticing that cigarettes self-extinguish largely mirrored implementation of RIP safety standards, this was not found during wave 4 in Canada. This finding may be because the RIP standard was set just before fieldwork began in October 2005 and it is likely many cigarettes were not yet compliant with the standard at the time of surveying. Only cigarettes manufactured after the implementation date were required to comply with RIP standards and it can take months for all old stock to be distributed and sold. Thus, it is possible that because the standard was so new, few respondents had significant exposure to the compliant cigarettes at the time of fieldwork. As would be expected, by wave 5 the percent of Canadian smokers reporting self-extinguishment and the frequency with which cigarettes self-extinguish increased dramatically. During wave 8 smokers in the US were significantly more likely than smokers in the other countries to report that their cigarettes self-extinguish and self-extinguish often. This may be, in part, due to the increased awareness of RIP standards in the US relative to the other countries because of the publicity attached to the state by state adoption of the RIP standard across the US. Additionally, it is possible that the required FSC marking on packs increased overall awareness. This latter explanation is unlikely as in Australia most packs have a statement about compliance in the 10% of the back of the pack not taken up by mandated health warnings, so smokers should be aware of it through this mechanism, even though there has not been a lot of other promotion.

These findings are tempered by a number of limitations. First, all data were based on self-report, which may be subject to social desirability and memory bias [24]. However, given that the question on awareness of cigarette self-extinguishing is not of a sensitive nature and being current smokers, their responses should be reasonably accurate. Second, we could not confirm whether the brand a given smoker used at the time of each survey wave was compliant with the law. In addition, it is unclear whether contraband cigarettes are RIP compliant or the extent to which respondents may have been using contraband cigarettes. Our data allowed us to only examine whether the law was in effect in the area of residence at the time of the survey and we were unable to control for other potential confounding factors (e.g. smoking history), so this may introduce error.

The present data suggest that introduction of RIP safety standards for cigarettes was not associated with changes in the number of cigarettes smoked per day; however, it may have influenced quit intentions. These data provided mixed findings for the impact of the law on consumer acceptability. However, while it is possible the tobacco industry could lose market share because the cigarettes may increase the desire to quit this would also lead to a positive net public health effect. Future research should assess whether quit intentions related to RIP safety standards actually result in quit attempts and successful quitting behaviors.

Overall, the RIP safety standards largely did not influence consumer acceptability. In addition implementation of these standards in jurisdictions may drastically reduce the number of cigarette related fires and the associated deaths and damages. Further research should assess how implementation of RIP safety standards has influenced smoking-related fire incidence, deaths, and other costs associated smoking-related fires. At present there is some evidence to suggest that fire deaths have substantially been reduced in Finland (http://www.firesafercigarettes.org.uk/news). Also, according to the National Fire Protection Association, fire deaths have dropped in the US despite increases in smoking among people covered by the law (http://www.nfpa.org/newsReleaseDetails.asp?categoryid=488&itemId=49272&rss=NFPAnewsreleases&cookie%5Ftest=1).

## Abbreviations

ASTM: American society for testing materials; CPD: Cigarettes per day; FSC: Fire standards compliant; GEE: Generalized estimating equations; RIP: Reduced ignition propensity.

## Competing interests

The authors declare that they have no competing interest.

## Authors’ contribution

GF and RB contributed to the design of the study and manuscript revisions. RJO conceptualized the analysis and provided manuscript revisions. SA performed the analysis, wrote the first draft, and revised the manuscript. HY KMC DH contributed to manuscript revisions. All authors read and approved the final manuscript.
